# Editors’ Book Table

**Published:** 1873-08

**Authors:** 


### Editors' Book Table.

[NOTE.—All works reviewed in the columns of the CHICAGO MEDICAL JOURNAL may be found in the extensive stock of W. B. KEEN, COOKE & CO., whose catalogue of Medical Books will be sent to any address upon request.]

BOOKS RECEIVED.

*A Guide to Urinary Analysis, for the use of Physicians and Students.* By HENRY G. PIFFARD, Physician to the Charity Hospital—to the New York Dispensary for Diseases of the Skin, etc., etc. William Wood & Co. 1873.

An excellent monograph, comprehending in less than a hundred pages what is most essential for the busy practitioner to know upon this much neglected subject. This book is an effort in the right direction, *i. e.*, to condense medical literature and simplify scientific proceedings. The subject-matter of the work appears to be fully up to the times, and the style and language far ahead of them. Appended is an index of the recent bibliography of urinary analysis which must prove a great assistance to students and writers in the prosecution of their investigations. The custom of appending to scientific works a bibliographical index is one which should be adopted universally. We hope to see the example set by the few followed by all. W. H.

*Manual of Thermometry*, for Mothers, Nurses, Hospitalers, etc., and all who have charge of the Sick and of the Young. By EDWARD SEGUIN, M.D. New York. Geo. P. Putnam & Sons. 1873.

This little volume is a condensed summary of the principles which underlie the relations of temperature to disease, derived in part from the author's own experience, and in part from the writings of Wunderlich, whose "Manual of Medical Thermometry" was translated by this author in 1871. The work is destined to prove valuable not only for what it actually contains, but also for many suggestions which will serve as initial points for more extended researches. The matter is good (we wish we could say as much for the manner of its presentation), the style is obscure, the language stilted and pedantic. The intermingling of sound medical science with doubtful theology is, to say the least, in very bad taste; more especially as, in his denunciation of what he terms "theurgic medicine," the author betrays the source from which his own theological (?) instructions have been derived—the Commune. *Ne sutor ultra crepidam.* W. H.

*An Introduction to the Study of Clinical Medicine, etc., etc.* By OCTAVIUS STURGES, M.D., Cantab., etc., etc.

The author of this "Introduction" has endeavored to teach the student, not so much what to observe, which he is supposed to learn from systematic text-books, but how to observe it, and has succeeded well in his endeavor. The work will assist the careful student in formulating his knowledge acquired empirically, and will amply repay a thoughtful perusal of its pages. If the reader learns nothing new therefrom, he will at least be taught the practical and useful application of much that he already knows, but had hitherto failed to utilize from lack of knowing how.

W. H.

*Chemistry: General, Medical, and Pharmaceutical, etc.; a Manual, etc.* By JOHN ATTFIELD, Ph.D., F.C.S., etc. 5th Ed. Henry C. Lea, Philadelphia. 1873.

One of the most useful and valuable hand-books within reach of the American medical student. An objection justly made to many of our text-books, and especially applicable to those of which chemistry is the subject, is their assumption of the possession of a greater amount of knowledge by the student than he can, in the majority of cases, legitimately claim. The evil consequences of this are apparent in the superficial character of the knowledge of chemistry acquired by most medical men. As a rule, the student comes to the study of his profession profoundly ignorant of the elements of the sciences which he is expected to study; unless, therefore, these sciences be presented to him, at first, in their simplest forms of expression, he will inevitably become discouraged, and instead of a savan, become a sciolist. To those who desire to understand chemistry, we cordially recommend Mr. Attfield's book, and would comprehend in this class, not only medical men, but all those who desire to gain an insight, from a scientific standpoint, into what is popularly termed "the chemistry of every day life." W. H.

*On the Treatment of Diseases of the Skin:* with an Analysis of Eleven Thousand Consecutive Cases. By Dr. McCall Anderson, Professor of Practice, etc., etc. Philadelphia: Henry C. Lea. 1873.

Dr. Anderson's analysis is valuable inasmuch as it is essentially practical, and especially so, that the author has evidently aimed to simplify the treatment of this troublesome class of maladies, paying much more regard to the substantial conditions of the case than to minute specification. His classification of skin diseases presents some new features well worth the attention of practitioners. The appearance of this work indicates progress in the right direction, *i. e.*, of simplicity rather than complexity in the study of disease. W. H.

*The Mechanism of the Ossicles of the Ear and Membrana Tympani.* By H. Helmholtz, Professor of Physiology in the University of Berlin. Translated from the German, with the author's permission, by Albert H. Buck, and Normand Smith, of New York. William Wood & Co., New York. 1873.

Helmholtz' name has become one of the watchwords of scientific progress in the present decade. The volume whose title is given above, is another addition to the many valuable contributions to science of this great German physicist and physiologist, whose labors have always been directed to the harmonizing of natural laws—the unification of science.

The present era in medical history, more than any preceding, is marked by the appearance of scientific monographs, evidences of the impetus which special studies have received, in obedience to the demands of the spirit of the age for greater scientific accuracy, and points of departure for new and more minute investigations. The student accepts eagerly and studies earnestly the little monograph which promises, at the cost of a few shillings in money, and an hour or two of study, to acquaint him with the facts and principles which he seeks to know, while he turns away in despair from the ponderous octavo in which the grain of wheat is to be reached by the purchase and sifting of the bushel of chaff. W. H.

